# Mechanical deconditioning of the heart due to long-term bed rest as observed on seismocardiogram morphology

**DOI:** 10.1038/s41526-022-00206-7

**Published:** 2022-07-12

**Authors:** Bradley Hoffmann, Parastoo Dehkordi, Farzad Khosrow-Khavar, Nandu Goswami, Andrew P. Blaber, Kouhyar Tavakolian

**Affiliations:** 1 University of North Dakota School of Electrical Engineering & Computer Science, Grand Forks, ND USA; 2 Heart Force Medical Inc, Vancouver, Canada; 3 Physiology Division, Otto Loewi Research Center for Vascular Biology, Immunology and Inflammation, Medical University of Graz, Graz, Austria; 4 Department of Biomedical Physiology and Kinesiology, Simon Fraser University, Burnaby, Canada

**Keywords:** Physiology, Anatomy, Arterial stiffening

## Abstract

During head-down tilt bed rest (HDT) the cardiovascular system is subject to headward fluid shifts. The fluid shift phenomenon is analogous to weightlessness experienced during spaceflight microgravity. The purpose of this study was to investigate the effect of prolonged 60-day bed rest on the mechanical performance of the heart using the morphology of seismocardiography (SCG). Three-lead electrocardiogram (ECG), SCG and blood pressure recordings were collected simultaneously from 20 males in a 60-day HDT study (MEDES, Toulouse, France). The study was divided into two campaigns of ten participants. The first commenced in January, and the second in September. Signals were recorded in the supine position during the baseline data collection (BDC) before bed rest, during 6° HDT bed rest and during recovery (R), post-bed rest. Using SCG and blood pressure at the finger, the following were determined: Pulse Transit Time (PTT); and left-ventricular ejection time (LVET). SCG morphology was analyzed using functional data analysis (FDA). The coefficients of the model were estimated over 20 cycles of SCG recordings of BDC12 and HDT52. SCG fiducial morphology AO (aortic valve opening) and AC (aortic valve closing) amplitudes showed significant decrease between BDC12 and HDT52 (*p* < 0.03). PTT and LVET were also found to decrease through HDT bed rest (*p* < 0.01). Furthermore, PTT and LVET magnitude of response to bed rest was found to be different between campaigns (*p* < 0.001) possibly due to seasonal effects on of the cardiovascular system. Correlations between FDA and cardiac timing intervals PTT and LVET using SCG suggests decreases in mechanical strength of the heart and increased arterial stiffness due to fluid shifts associated with the prolonged bed rest.

## Introduction

The human cardiovascular system has evolved to operate in the presence of gravity^1–3^ When standing on Earth, hydrostatic gradients reduce arterial pressures located above the heart, while also increasing pressures below the heart, which induces local arterial responses^4,5^ When introduced to weightlessness, the physical unloading and lack of force pulling blood to the lower extremities causes the phenomenon of upward fluid shift^6^. The once unequal gradient pressures in blood vasculature now equalize, affecting blood pressure regulation and cardiovascular control^7–9^ Seen in both short and long-term spaceflight, upward fluid shifts have been associated with increased orthostatic intolerance post flight^10–13^.

Continuing effects of physical unloading in microgravity can drive cardiovascular remodeling and arterial changes leading to mechanical attenuation of heart function and advanced arterial stiffness^14–16^. There are interventions being investigated to act as countermeasures to stem the physiological deconditioning of spaceflight (i.e., lower body negative pressure (LBNP) application, exercise activities, short-arm centrifugation, plasma volume replenishment, and nutrient supplementation)^7,17–21^, However, little is known about the effects of extended weightlessness on arterial stiffness and systemic vascular resistance. Hughson et al. showed an increase in arterial stiffness of astronauts after six months aboard the International Space Station similar to that of 10–20 years of aging^22^. The arterial stiffness increase was based on observations of decreased pulse transit time (PTT) and lowered biomarkers, such as insulin^22^. Pulse wave transit time has also been reported to be decreased even after 5 days of spaceflight^23^.

A major limiting factor in the interpretation of spaceflight data is the relatively low sample size of individuals who have experienced spaceflight. Therefore, conditions such as 6° head-down tilt bed rest are routinely used as space analogs to simulate the effects of microgravity^24–26^ Head down tilt bed rest has been shown to mimic the effects of weightlessness on the body including upward fluid shifts^27^. Several studies indicate that vascular remodeling after long-duration bed rest produces sustained decrease left ventricular mass during bed rest while causing drastic deconditioning of left ventricular volumes^24,28–34^, although, a recent study found that ventricular mass loss did not occur^35^. However, in spaceflight, there is a chronic decrease in left ventricular mass of values close to 9–12% loss while in similar observations during HDT studies show 8–16% mass losses^29,30,36^, Alternatively, decreases in left ventricular volumes have been attributed to blood plasma loss during bedrest also seen in spaceflight^27,35^. In tandem to ventricular remodeling, responses of blood pressure have been shown to lower during head-down tilt bed rest (HDT) analogous to spaceflight^37–39^.

The current study focused on the mechanical performance of the heart during 60-day HDT. With each heartbeat there are mechanical events that give a windowed look into cardiac performance. Cardio-mechanical techniques such as, Seismocardiography (SCG), evaluated via a local accelerometer placement on the chest, can measure cardiac motion, giving information about heart valve opening and closure events^40^. Techniques used to measure cardio-mechanical vibrations have been used to observe deconditioning of the cardiovascular system in spaceflight and early hemorrhage detection^41–44^.

In development of a smart garment, Di Rienzo et al. utilized left ventricular ejection time (LVET) and QS2 (electromechanical systole, relation of SCG-AO and ECG Q-wave) as measurements of heart contractility^42,45^. Another study by Di Rienzo et al., used SCG techniques for monitoring vital signs during sleep of astronauts on the ISS^46^. In that study, the group used the smart garment with a three-axis SCG on the sternum and a three-axis gyroscope to gather cardiovascular vibrations on the chest and evaluate cardiovascular data during sleep. Initial analysis was done on one astronaut crew member using timing intervals of isovolumic contraction time (ICT), isovolumic relaxation time (IRT), LVET, and pre-ejection period (PEP) over seven sleep intervals^46^.

Detection of the effects of early-stage hemorrhage have also been investigated through using SCG. Tavakolian et al., investigated simulated hemorrhage through graded LBNP to quantify correlations between stroke volume via echocardiography and SCG features^43^. Of the features derived by SCG, timing intervals of LVET and PEP were shown to be highly correlated to changes during graded LBNP. This correlation suggested that changes of SCG-derived features (e.g., LVET) in emergency scenarios can be used as warning signs of early hemorrhage^43^.

This investigation utilized the HDT space analog together with cardio-mechanical responses of the heart to further the understanding of cardiovascular compliance and resultant arterial stiffness. The cardiovascular vibration technique of SCG was used to provide insight into the mechanical deconditioning of the heart through relationships between blood pressure^47^ and cardiovascular timing intervals^43,48.49^. It was hypothesized that with increased headward fluid shifts during HDT there would be a decrease in SCG amplitude strength due to mechanical deconditioning of the heart. As a secondary hypothesis, it was predicted that PTT would decrease due to increased arterial stiffness associated with HDT.

## Methods

### Bed rest protocol

The head-down tilt bed rest study consisted of two campaigns of 10 volunteers each lasting 60 days. Campaign 1 (height = 1.76 m ± 0.06, weight = 74.86 kg ± 7.81) was started in January 2017 while Campaign 2 (height = 1.76 m ± 0.04, weight = 73.10 kg ± 7.05) was started in September 2017. The clinical trial was registered at ClinicalTrials.gov (Identifier: NCT03594799). An all-male cohort (N = 20) participated in this experiment (ages ranging from 20–45). The experimental group consisted of nutrient countermeasure group randomly selected (N = 10) and control group (N = 10). This study data collection followed in-line with our previous study of cardio-postural effects of prolonged bed rest^37^. The experiments were conducted as an ESA funded study at the Institut de Médecine et de Physiologie Spatiales (MEDES), a Centre National d’Études Spatiales (CNES) facility located in Toulouse, France. This prolonged bed rest study was broken into three phases. Phase one consisted of 14 days of baseline data collection (BDC) prior to head down tilt, phase two consisted of 6-degree head-down tilt (HDT) bed rest for 60 days and phase three was a recovery period (R) 14 days after head-down tilt. Ethical approval was obtained from the Comité de Protection des Personnes / CPP SudOuest Outre-Mer I and the Agence Française de Sécurité Sanitaire des Produits de Santé for each facet of the experimental protocols. Additionally, approval of this study was obtained by the Office of Research Ethics at Simon Fraser University. The participants signed a written informed consent prior to taking part in the study.

Data collection days and times were selected to avoid conflict with the ESA orthostatic tolerance testing. In the previous study protocols, supine to stand (STS) were used to assess the relationship of cardiovascular control and posture^37^. Two of the STS collections were taken during baseline before bed rest (BDC) and post bed rest recovery (R). The data collection for STS was taken at the same time on BDC12 (12 days before) and BDC2 (2 days before) for baseline before bed rest. Data collection of STS after bed rest was taken at R8 (8 days after bed rest). STS data collection consisted of 5 min of collection in supine and 6 min of data collection during standing. In this investigation, only the supine portion of STS testing was investigated for cardiovascular function (ECG, blood pressure, and SCG). During HDT, the cardiovascular function was assessed at 6 degrees head-down for 10 min via ECG, blood pressure, and SCG at the same time in the morning on days of HDT 1, 29 and 52 (days during head-down tilt). Plasma volume was measured using CO rebreathe and were made as part of the standard bed rest protocol used by ESA^24^.

### Signal acquisition

**Fig. 1:**
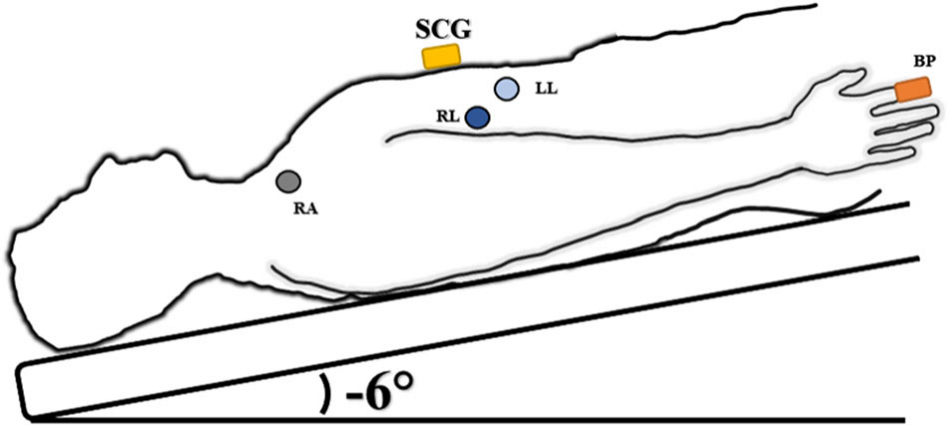
HDT schematic of sensor placement. SCG (yellow rectangle) placed on the xiphoid process. Blood pressure measured at the finger (orange rectangle). ECG Lead II shown RA lead (gray circle) on right clavicle, RL lead (dark blue circle) on lower right rib cage and LL (light blue circle) on lower left rib cage.

Continuous blood pressure was collected via non-invasive Portapres (FMS, Amsterdam, The Netherlands). SCG was collected by a unidirectional accelerometer in the dorso-ventral direction positioned on the xiphoid process of each subject. The SCG measured the vibrations of the heart as a resultant beat against the chest wall during each cardiac cycle. Electrocardiogram (ECG) was collected using three lead ECG positioned in a Lead II configuration (FD-13, Fukuda Denshi Co. Ltd, Tokyo, Japan). Experimental setup shown in HDT schematic (Fig. 1). A sampling rate of 1,000 Hz was used for data gathering through National Instruments USB-6218 16-bit data acquisition system and using LabVIEW 2013 software (National Instruments Inc, TX, USA).

### Data analysis

**Fig. 2:**
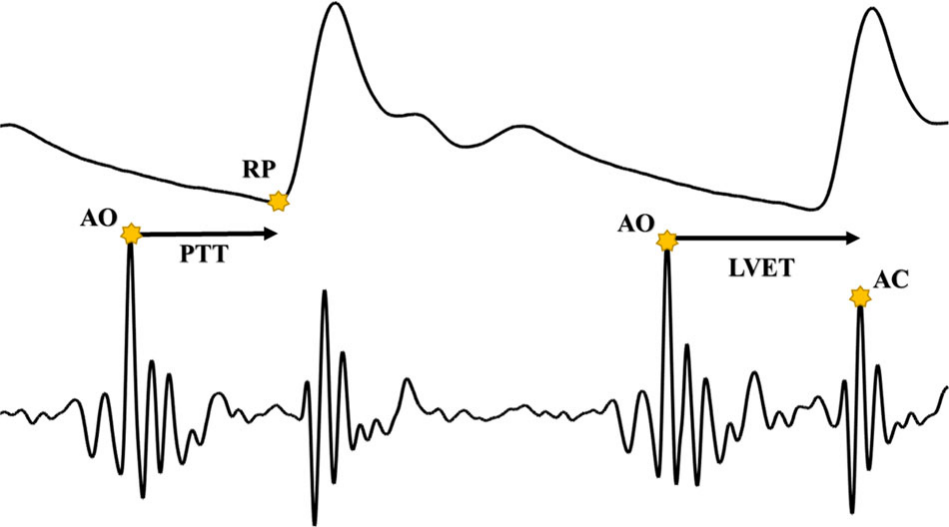
Blood pressure and seismocardiogram waveforms with annotations. Pulse Transit Time (PTT) is the time interval between the aortic valve opening (AO) peak of SCG and RP (Foot) of BP. LVET is the time interval between the AO and AC peak of the SCG.

The fiducial points of AO (aortic valve opening) and AC (aortic valve closing) were annotated on SCG^49,50,51^, The cardiovascular timing feature PTT was collected as the timing between the AO peak of SCG and the foot feature of the blood pressure waveform^48^ (Fig. 2). Additionally, the left-ventricular ejection time (LVET) was measured as the time interval between SCG-AO and SCG-AC.

Beyond individual fiducial points, the entire morphology of SCG was analyzed by functional data analysis (FDA) using MATLAB R2019a^52^. Similar techniques have been used in analysis and interpolation of SCG fiducial points^53–55^ In FDA, each SCG cycle was modeled as the linear combination of 23 spline base functions of order 4. The coefficients of the model were estimated and compared over 20 cycles of SCG recordings between BDC12 and HDT52 for the 20 subjects.

### Statistics

Nutrient countermeasure was randomized amongst participants. Statistical analysis of Cocktail countermeasure followed the same technique as outlined in previously reported^37^. If no significance was found between control and countermeasure group, then the participants were merged for further analysis. Normality test was conducted using Shapiro-Wilk at α = 0.05.

For normally distributed data, two-way ANOVA with replication was completed followed by a Bonferroni correction comparing cardiovascular changes due to bed rest between BDC12 compared to HDT01, HDT29, HDT52 and R8 in addition to campaign 1 and campaign 2 for seasonal differences. For non-normally distributed data a nonparametric test was conducted using the Friedman test. A probability of α < 0.05 was considered significant. Additionally, the Wilcoxon Signed Rank test was performed to evaluate the differences between FDA coefficients for BDC12 and HDT52, to determine SCG morphology changes.

## Results

As previously reported by Xu et al.^37^, the cocktail countermeasure had no effect on the cardiovascular values, and our analyses indicated that this was also the case for the timing intervals in this study (PTT, *F* < 0.01, *p* = 0.998 and LVET, *F* = 0.47 *p* = 0.495).

### SCG morphology and functional data analysis

**Fig. 3:**
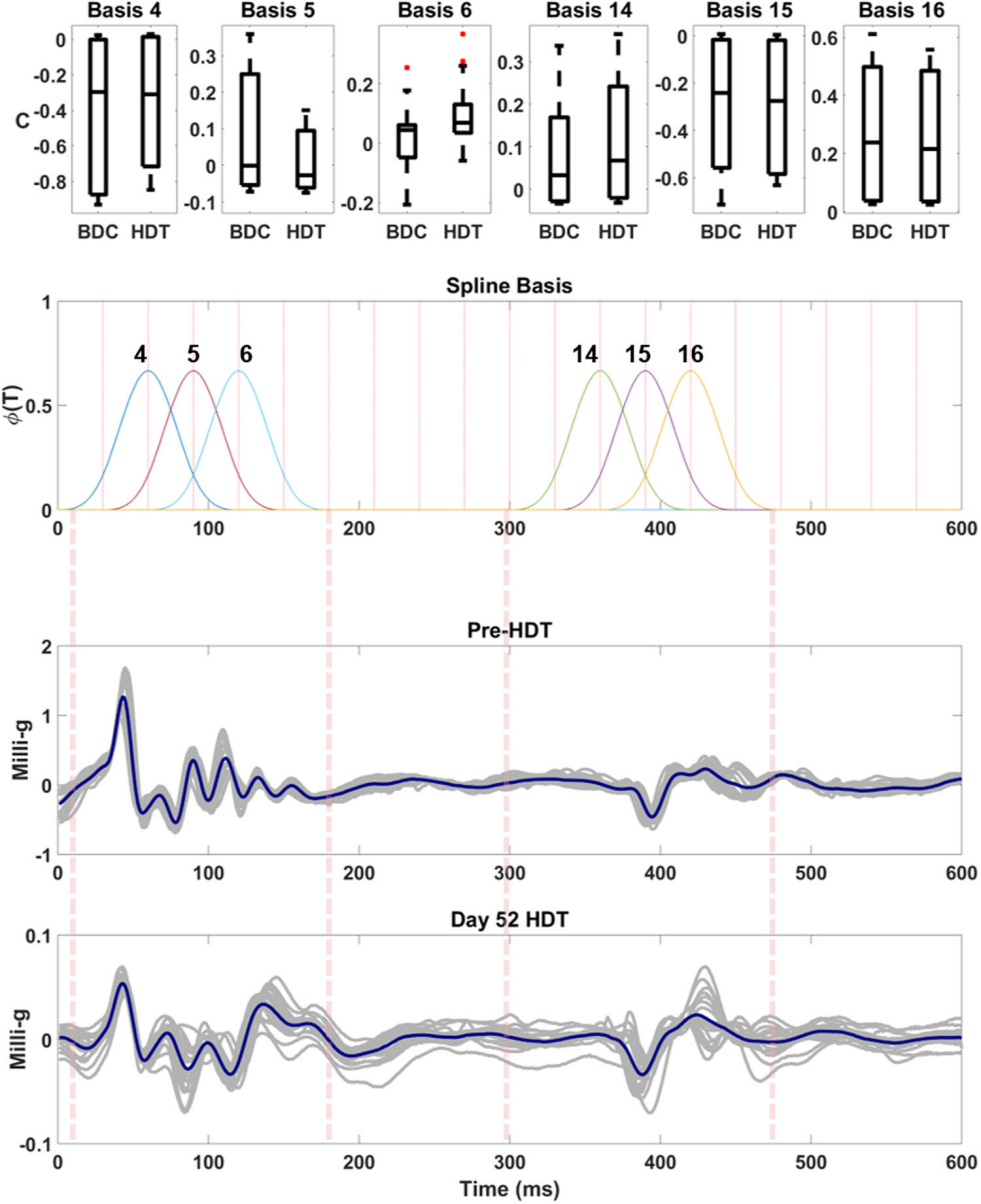
FDA analysis of SCG signals containing AO and AC basis function coefficient sets pre- and post-HDT. Average AO and AC peak decrease over 20 cardiac cycles after 52 days head-down tilt bed rest compared to pre-HDT. Morphology spline coefficient sets corresponding to the AO (sets 4, 5 and 6) and AC (sets 14, 15, and 16) peak complexes show distributions from pre-HDT to day 52 HDT furthering the peak changes. Coefficient sets define the spline basis functions shown to describe the SCG signal morphology. Boxes represent ±1 interquartile range, whiskers represent ± 1.5 interquartile range and center lines are medians.

The heart was analyzed via the vibration peaks caused by the heart hitting the chest wall during each beat. These vibrations morphologies AO and AC detected at the xiphoid process by the SCG showed a lowering trend through bed rest. Through FDA, the coefficients of corresponding spline basis functions describe the SCG waveform in 23 knots. These knots act as windows of the signal dividing it into sections defined by the specific splines shown to represent SCG morphology. The coefficient sets showed wider distributions at the end of bed rest compared to the baseline data collection sets. Spline basis coefficient sets 4, 5 and 6 describe the AO peak complex, while Spline coefficient sets 14, 15, and 16 describe the AC peak complex (Fig. 3). The values of AO and AC exhibited mixed behavior and Friedman test was conducted. Averages of 20 cardiac cycles across all 20 subjects showed a decrease in peak morphologies of both AO and AC complexes pre-HDT to day 52 of HDT. Furthering the changes in morphology the peak distribution of AO and AC showed changing allocation of coefficients due to decreasing peak complex amplitudes towards the end of bed rest.

### Prebed rest to postbed rest cardiovascular responses

**Fig. 4:**
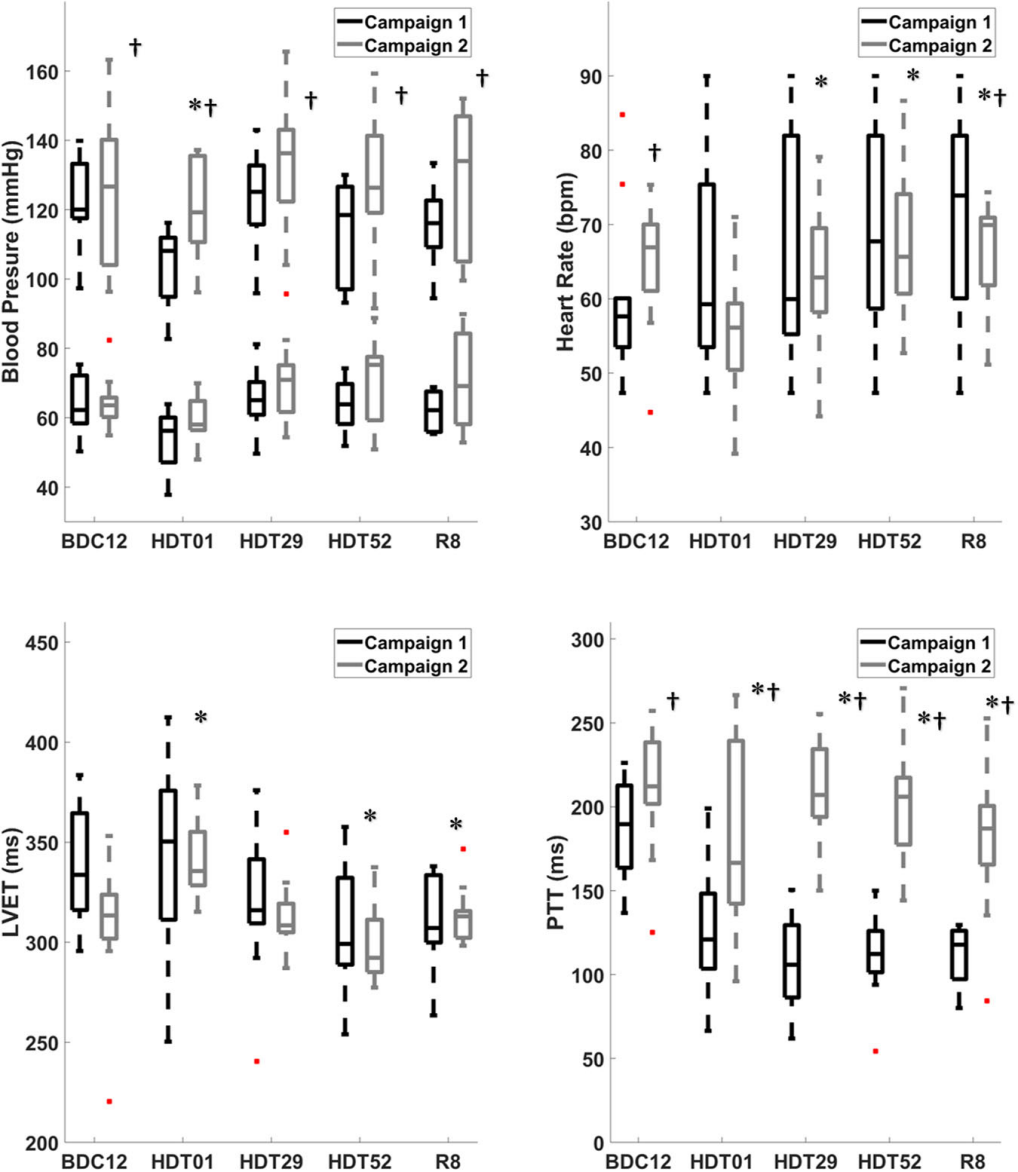
Cardiovascular function through the three phases of bed rest. Cardiovascular timing intervals of PTT and LVET were taken from the relationships of SCG and show decreasing trends. PTT has a drastic average decrease that does not recover after 8 days post bed rest. LVET has a variable adjustment to fluid shifts of bed rest but decreases towards the end with a slight recovery. Blood pressure values adjust to fluid shifts with an initial decrease but stabilize towards the end of bed rest. Upper values in the plot represent systolic BP and lower values, diastolic BP. Boxes represent ± 1 interquartile range, whiskers represent ± 1.5 interquartile range and center lines are medians. * Denotes significant differences compared to BDC12 and † denotes significance between campaigns at each test day (*p* < 0.05).

Cardiovascular timing intervals measured from the SCG peak vibrations were shown to be affected by prolonged bed rest (Table 1). Values of HR, DBP, SBP, MAP, PTT and LVET passed a test of normality (*p* > 0.05). PTT from the AO peak of SCG to the foot of the blood pressure at the finger, revealed a drastic decrease immediately into bed rest for both campaigns (Fig. 4). On HDT day 1, PTT fell dramatically and stabilized significantly faster than baseline towards the end of bed rest on day 52 HDT (*p* < 0.01). The PTT interval did not recover by R8. Additionally, there were significant differences between the campaigns (*p* < 0.01). PTT decrease more drastically between pre- to post-bed rest in Campaign 1 compared to Campaign 2 (Fig. 4).

**Table 1:** Cardiovascular function and timing intervals through the three phases of bed rest.

Variable	BDC12		HDT01		HDT 29		HDT52		R8	
	Campaign 1	Campaign 2	Campaign 1	Campaign 2	Campaign 1	Campaign 2	Campaign 1	Campaign 2	Campaign 1	Campaign 2
HR (bpm)	55 ± 4	64 ± 8^†^	59 ± 11	55 ± 10	61 ± 13^*^	63 ± 9^*^	66 ± 15^*^	67 ± 11^*^	71 ± 14^*^	67 ± 7^*†^
RR (ms)	917 ± 67	1067 ± 133^†^	983 ± 183	917 ± 167	1017 ± 217^*^	1050 ± 150^*^	1100 ± 250^*^	1117 ± 183^*^	1183 ± 233^*^	1117 ± 117^*†^
SBP(mmHg)	118 ± 13	125 ± 21^†^	104 ± 11^*^	120 ± 15^*†^	123 ± 15	132 ± 18^†^	113 ± 15	128 ± 19^†^	116 ± 11	129 ± 21^†^
DBP (mmHg)	63 ± 8	65 ± 7	54 ± 8^*^	59 ± 7^*^	66 ± 9	71 ± 12	64 ± 8	71 ± 12	62 ± 5	70 ± 13
MAP (mmHg)	83 ± 10	85 ± 12	71 ± 9	79 ± 10	85 ± 10	91 ± 13	80 ± 9	90 ± 14	80 ± 7	90 ± 15
PTT (ms)	189 ± 29	209 ± 38^†^	123 ± 40^*^	182 ± 60^*†^	106 ± 29^*^	208 ± 31^†^	110 ± 26^*^	205 ± 36^†^	113 ± 17^*^	179 ± 45^*†^
LVET(ms)	337 ± 28	308 ± 35	344 ± 49^*^	340 ± 21^*^	320 ± 38	312 ± 20	308 ± 34^*^	300 ± 20^*^	310 ± 23^*^	313 ± 15^*^

Campaign groups were paired comparing HDBR phases to baseline BDC12. Further unpaired analysis was done to compare Campaign 1 and Campaign 2. Cardiovascular timing intervals were Values are split based on phase and further split based on campaign season. Campaign 1 coincides with the first season started in January and Campaign 2 coincides with the second season started in September. * Denotes significant differences compared to BDC12 and † denotes significance between campaigns at each test day (Two-way ANOVA, p < 0.05).

LVET had an initial increase on HDT01 with a further decrease from HDT29 to HDT52. The value for LVET showed a slight recovery on R8 but remained significantly lower than on BDC12 (*p* < 0.01). This trend was seen in both campaigns; however, like PTT, there was a significantly greater change of LVET between campaigns 1 and 2 (Fig. 4).

Heart rate (HR) had a gradual increase through the entirety of bed rest and 8 days post HDT on R8 (*p* < 0.03) for both campaigns. Campaign 2 had less of an increase in HR compared to campaign 1, significant differences at BDC12 and R8. Systolic blood pressure (SBP), diastolic blood pressure (DBP) and mean arterial pressure (MAP) showed significant changes across bed rest phases (*p* < 0.04). Additionally, there were large significant differences in these changes between campaigns 1 and 2 in blood pressure values. Through bed rest, campaign 1 had lower systolic (*p* < 0.001) and diastolic blood pressures compared to campaign 2 (Fig. 4). Blood plasma volume decreased an average of 19% from BDC12 (4.10 ± 0.51 L) to the end of HDT (3.31 ± 0.37 L), (*p* < 0.03). No significant change was seen between campaigns (*p* = 0.45). Additionally, fitness levels (V̇O_2_max) of the participants when they entered the study (BDC 8 baseline) were not different between campaign 1 (39 ± 4 ml/min/kg) and 2 (40 ± 4 ml/min/kg). Fitness decreased by similar amounts in both campaigns to of 31 ± 4 ml/min/kg (R1, campaign 1) and 29 ± 2 ml/min/kg (R1, campaign 2).

## Discussion

In this study, the timing interval of pulse transition time decreased analogous to that seen in 6-months of spaceflight^22,23^, This decrease in PTT of 15–40% without recovery suggests that during 60-days head down tilt there are similar mechanics producing a decrease in vascular compliance leading to increases in arterial stiffness. Likewise, decreases in LVET and attenuation of the SCG peak vibrations compared pre to postbed rest suggests a decrease in mechanical performance of the heart due to upward fluid shifts.

Furthermore, differences in blood pressure values and cardiovascular timing intervals between bed rest campaigns suggests a seasonal influence acting on the cardiovascular system. This study, to the best of our knowledge, is the first investigation using mechanical vibration techniques of SCG to derive cardiovascular timing intervals during prolonged HDT. Additionally, this is the first investigation to bring attention to possible seasonal influences on cardiovascular function during prolonged HDT.

The morphology changes observed with SCG during HDT bed rest supports our hypothesis of mechanical attenuation of the heart vibrations associated with headward fluid shifts. Additionally, this attenuation of SCG was concurrent with a decrease of PTT which supports the secondary hypothesis of increased arterial stiffness through prolonged bed rest. The underlying mechanism that has the highest affect upon the mechanical deconditioning could be related to the vascular changes due to headward fluid shifts in the body. However, headward fluid shifts and changes in hydrostatic pressure together with the lack of compression on the chest has been shown to increase the geometry of the thoracic cage^25^. Dampened vibrations could also occur due to expansive distension of the thorax as another factor in this vibrational attenuation.

Cardiovascular vibrational assessment techniques have been used in a multitude of previous studies. One such technique parallel to SCG is that of ballistocardiography. Ballistocardiography can record recoil ballistic forces which occur as blood is ejected into the vasculature can be measured through multiaxis sensor placement on the body,^56^. One recent study by Rabineau et al. discusses the effects of exercise countermeasure as a mitigation strategy for cardiovascular deconditioning during bed rest^57^. In their finding, ballistocardiography (6-degree of freedom) and apex-SCG were used as monitoring techniques of kinetic energy instead of vibrational peaks and cardiovascular timing. These relationships were used to show the promise of a reactive jump exercise toward prevention of orthostatic intolerance. However, placement of the SCG beyond the traditional sternum placement creates a signal susceptible to noise artifacts^57,58^.

Vibrational signals are dependent on mass components of the system, defining the equations of motion. In addition to increased upward fluid retention, systemic loss of cardiovascular mass can contribute to the lowered mechanical peak performance. In spaceflight, there is a chronic decrease in left ventricular mass of values close to 9–12% loss while a similar observation during HDT studies show 8–16% mass losses^29,33.36^, Ventricular mass losses have also been observed in previous bed rest studies^34^. These changes in mass produce hinderances in both left ventricular end-systolic volume and end-diastolic volume. However, vascular changes and pressure regulatory responses to fluid shifts cause relatively quick recovery stabilization of the left ventricular end-diastolic volume^29,59^, while left ventricular end-systolic volume tends to continue to increase during prolonged weightlessness suggesting a reduction in cardiovascular compliance and functional performance^60^.

As shown in the decreased averages and distributions of the FDA spline basis coefficient sets, there are displays of underlying structural changes in SCG peak complexes (Fig. 4). Upon entrance into HDT01, instantaneous decrease of the SCG peaks occur due to the blood displacement towards the head. The spline basis function coefficient distributions define a lowering trend of both AO and AC structures towards the end of bed rest. Structural decreases in these SCG peak vibrations suggests there is a prolonged deconditioning that occurs potentially as seen in ventricular mass loss and increased headward fluid volumes.

During spaceflight and prolonged bed rest, regulatory responses of blood pressure occur as the body attempts to retain homeostasis. The shift in blood pressure throughout bed rest is shown in the increase in HR and fluctuational decreases of LVET and PTT. The observation resulting in instantaneous increase in LVET upon commencement of HDT is in correlation between this initial regulation of the increased upward blood volume. Initial increases of central blood volumes seen in head-down tilt bed rest, as well as weightlessness of microgravity cause increased stroke volume and increased cardiac output^8,14^, Resultant increases in LVET at HDT01 seen in our study are defined by these decreases of blood pressure which could be a response to increased stroke volume (Fig. 4). However, as HR remains continually higher the resultant mechanical deconditioning of the left ventricle causes the continual decrease of LVET. As bed rest confinement continues, offloaded conditions and headward fluid shifts had a prolonged effect on LVET values that showed significant decreases at the end of HDT. The relationship between potential stabilizing left ventricular end-diastolic volume with increased left ventricular end-systolic volume in conjunction with lowered offloaded conditions of bed rest could be a major contributing factor for the decrease of LVET. As seen in previous literature, left ventricular end-diastolic volume and end-systolic volume begin to recover quickly after weightlessness and HDT bed rest^32^. The quick recovery in these parameters could be a driving factor leading to the gradual recovery of LVET 8 days post bed rest seen in an increased value on R8. However, as there were small losses in amplitude peak strength of AO and AC, LVET recovery was relatively small.

The decrease of the pulse transit wave defined by PTT to the finger, is analogous to during 6-months of space flight seen by both Hughson et al and Baevsky et al., suggesting the presence of a resultant stiffening arteries during bed rest^22,23^, Upon commencement of HDT01, the PTT values show a decrease of 15–35%. Plasma volume had shown to decrease as well between baseline at BDC compared to the end HDT similar to that found by Pavy-le Traon et al., of blood plasma volume decreases of 10–15% in both spaceflight and head-down tilt bed rest. The viscosity changes of the blood due to this decrease could have potential factors effecting PTT shifts and SCG attenuation^27^. However, there were no significant differences in blood plasma volume between campaigns still suggesting seasonal changes have an effect on PTT during bedrest. As PTT decreased at HDT01, there was an initial decrease in SBP and DBP while HR increased that point to potential relaxation precursors of vaso-controlled responses of vasculature. Acute weightlessness and upward fluid shifts seen by Norsk et al. reduces vascular resistance if 24% while chronic weightlessness decreases vascular resistance by 14% suggesting a presence of vasorelaxation^14^. Prolonged bed rest towards HDT52 and 8 days post of recovery both show decreased PTT values of 15–40% without recovering hinting at altered vascular compliance. The altered vascular compliance could be in response to continued relaxation of blood vasculature to accommodate blood flow due to headward fluid shifts.

**Fig. 5:**
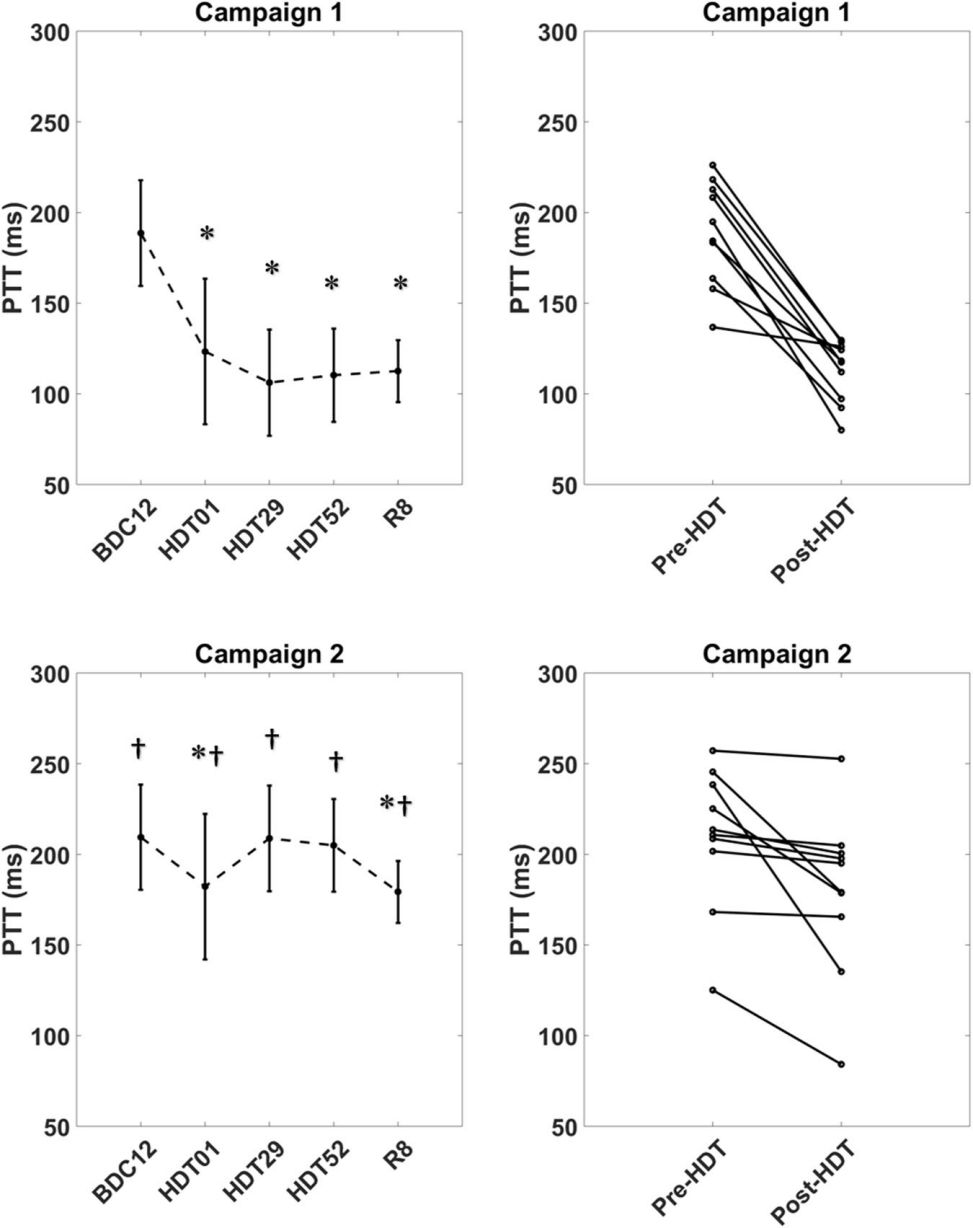
PTT comparison through bed rest phases and campaign differences. Rapidly quickening of PTT to the finger upon entrance into HDT through the end of bed rest suggesting increased arterial stiffness due to responses of fluid shifts. The value does not recover 8 days post bed rest. Due to seasonal temperature changes campaign 1 had shown more drastic decrease in pulse transit time due potentially to vascular vaso-controlled responses. Data is represented as mean ± standard deviation. * Denotes significant differences compared to BDC12 and † denotes significance between campaigns at each test day (*p* < 0.05).

As vascular adaptation occurs, the drop in hydrostatic pressure causes activation of regulatory responses of the baroreflex. The lowered force acting on the blood due to lack of gravity causes lowered shear stress between the blood and vasculature^24^. Vascular distension that occurs due to increased upward localized blood volumes causes increased arterial elasticity, a reduction in arterial compliance and stiffer arteries also seen in previous bed rest studies^24,31^, Rapidly increased headward blood volumes inhibit the ability of vasculature to expand quickly to allow absorbing vibrational effects of traveling blood, resulting in the quickening of PTT to the finger (Fig. 5). Reductions in arterial compliance along with a reduction in systemic vascular resistance (SVR) are compounding affects that continue to drive the decrease in the mechanical performance of the heart that is observed in both SCG peak complexes and cardiovascular timing intervals (i.e., LVET and PTT). Bed rest and fluid shifts have a significant impact on vasculature changes, of these impacts are increased arterial stiffness as seen in the dramatic decreases of cardiovascular timing intervals such as PTT.

Though both campaigns showed similar trends in cardiovascular relationships and timing intervals, there was a significant difference in the changes between the values over bed rest between campaigns. In campaign 1 as compared to campaign 2, blood pressure (SBP and DBP) values showed lower trends. These changes could potentially be attributed to the seasonal differences (that can influence vaso-control of vasculature) in the two campaigns. As campaign 1 was started in January, SBP in this season is expected to be higher due to constriction of vasculature for thermal regulatory responses^61,62^, Upon entering the bed rest study, the individuals would be in a higher indoor temperature environment compared to outdoor ambient temperatures, allowing for more dilation of vasculature to continue this thermal regulation. Previous literature has shown that by increasing both indoor and outdoor temperatures by 1 °C resultant blood pressure reduction occurs^61,63.64^, In campaign 2, which was begun in September, the differences in outdoor and indoor temperatures were not as drastic leading to a lessened vascular response (Fig. 4). The changes in blood pressure and vascular constriction or dilation further influence the heart rate. This was observed between the two campaigns: in campaign 1 there is a large increase in HR pre- to post-bed rest while in campaign 2, there is a smaller increase in HR during pre- to post-bed rest period. These differences in heart rate between the two campaigns could have contributed to the differences in PTT. Throughout bed rest, PTT shows more drastic decreases in campaign 1 due to fluid shifts (and perhaps thermal responses), where in campaign 2 PTT shows lesser decreases (Fig. 5). Between the two campaigns, fitness levels (V̇O_2_max) were not significantly different between baselines and recovery stages. Average values of campaign 1 and campaign 2 differed by only 1 ml/min/kg (campaign 1 BDC8 = 39 ± 4 ml/min/kg, campaign 2 BDC8 = 40 ± 4 ml/min/kg. Similarly, V̇O_2_max values for recovery (R) differed only by 2 ml/min/kg (campaign 1 R1 = 31 ± 4 ml/min/kg, campaign 2 R1 = 29 ± 2 ml/min/kg. The small variation of fitness level suggests that the differences in BDC HR were most likely related to seasonal influences rather than cardiorespiratory fitness.

Due to the influences of thermal regulatory responses, potential effects of seasonal changes should be considered in future bed rest studies. During prolonged spaceflight Stahn et al. investigated that core body temperature increases 1 °C which can have an effect on task performance^65^. Norsk et al. points to both cardiovascular shifts and thermal regulatory responses to decreases of systemic vascular resistance in prolonged spaceflight^25^. Our results from prolonged bed rest confinement suggest that training temperature of the astronaut and the environmental temperature during spaceflight could potentially influence the changes in cardiovascular function and/or cardiovascular responses. In addition, such temperature changes could further influence the impacts associated with upward fluid shifts and vascular remodeling during prolonged bed rest.

In this study, the participants involved were all males. It has, however, been shown that cardiovascular responses are influenced by sex^19,66,67^, Future studies should, therefore, include both males and females. Plasma volume loss was not directly studied in the scope of this investigation as a correlation to seasonal changes and cardiovascular timing. Future studies should investigate blood volume as a potential metric of cardiovascular and thermal strain. Another limitation of this study is that temperature was not controlled. As our results show, temperature changes could potentially affect several parameters. Future bed rest studies should take into account the effects of seasonal changes on cardiovascular and other responses.

During spaceflight, the cardiovascular system alone experiences rapid deconditioning due to vascular changes occurring during upward fluid shifts. Prolonged head-down tilt bed rest has shown to be analogous to the impacts seen in microgravity. This study has shown the loss of mechanical strength of the heart due to prolonged head down tilt bed rest. Our results supporting the hypothesis of continued attenuation of heart vibrations resulting from HDT. The physical-mechanical strength loss seen from the peak inflections of SCG in tandem with blood pressure responses, suggest that prolonged fluid shifts result in the quickening of cardiovascular timing intervals corresponding to vascular changes. The rapid drop in PTT had shown that immediately experiencing fluid shifts cause faster blood distribution transition times to the finger, resulting in the potential for increased arterial stiffness and lowered arterial compliance. Quickening LVET appears to be correlated to the decreases in mechanical strength of the left ventricle, which could arise due to changes in blood volume and ventricular mass loss associated with the HDT.

Our results show that seismocardiography can provide higher fidelity information about the mechanical performance of the cardiovascular system during prolonged HDT bed rest. As the heart beats, there are critical time elements that correspond to resultant vibrations. Traditional, techniques that are utilized to gather cardiovascular data overlook this mechanical stimulus and require complex instrumentation. Complex equipment can make monitoring difficult in both operational spaceflight tasks (e.g., extravehicular activity) or during routine medical checkups. Single sensor placement of the SCG can yield crucial cardiac information with less instrumentation and opens monitoring techniques to wearable technologies (e.g., shirt or bands) for spaceflight applications as well as home care monitoring. Insight gained from this study can be further used to gain understanding of how the vasculature and heart adapts mechanically to sedentary bed rest or simulated prolonged weightlessness.

Finally, The results of this study can give insight into the continuing cardiovascular changes due to aging as well as the consequences of bed rest confinement during hospitalization^39,68^, Not only can the use of analogs such as HDT build upon our understanding of spaceflight physiology, but they can also aid in terrestrial medical applications.

## Data Availability

The datasets generated during or analyzed during this study are available from the authors upon request.
